# Diabetes Mellitus among Patients with Non-alcoholic Fatty Liver Disease Visiting the Outpatient Department of Internal Medicine in a Tertiary Care Centre

**DOI:** 10.31729/jnma.8324

**Published:** 2023-11-30

**Authors:** Prabin Adhikari, Kaushal Oli, Saloni Shrestha

**Affiliations:** 1 Department of Internal Medicine, Nepal Medical College and Teaching Hospital, Jorpati, Kathmandu, Nepal; 2 Nepal Medical College and Teaching Hospital, Jorpati, Kathmandu, Nepal

**Keywords:** *diabetes mellitus*, *fatty liver*, *non-alcoholic fatty liver disease*, *prevalence*

## Abstract

**Introduction:**

Diabetes mellitus is a chronic metabolic illness which is mainly associated with reduced physical activity and obesity. Diabetes mellitus and non-alcoholic fatty liver disease may coexist and synergistically lead to poor clinical outcomes. The aim of the study was to find out the prevalence of diabetes mellitus among patients with non-alcoholic fatty liver disease in a tertiary care centre.

**Methods:**

A descriptive cross-sectional study was conducted among patients with non-alcoholic fatty liver disease in the outpatient department of Internal Medicine of a tertiary care centre between 1st January 2022 and 29th June 2023. Convenience sampling was done. A convenience sampling technique was used. The point estimate was calculated at a 95% Confidence Interval.

**Results:**

Among 150 patients with non-alcoholic fatty liver disease, diabetes mellitus was seen among 18 (12%) (6.80-17.20, 95% Confidence Interval). Diabetes mellitus was more prevalent in males 12 (66.67%) in patients with non-alcoholic fatty liver disease.

**Conclusions:**

The prevalence of diabetes mellitus among patients with non-alcoholic fatty liver disease was found to be lower as compared to similarly reported research studies.

## INTRODUCTION

Diabetes mellitus (DM) is a chronic metabolic illness mainly associated with reduced physical activity and obesity.^1^ Non-alcoholic fatty liver disease (NAFLD) is defined as more than five per cent of fat infiltration in the liver without alcohol consumption exceeding 30 grams/day in men and 20 grams/day in women. Diabetes mellitus and NAFLD can coexist and synergistically result in poor clinical outcomes.^2^

Non-alcoholic fatty liver disease may lead to lipotoxicity that can further give rise to insulin resistance and is a major risk factor for diabetes mellitus.^3,4^ There are very few studies conducted in Nepal regarding the prevalence of diabetes mellitus among patients with non-alcoholic fatty liver disease.

The aim of the study was to find out the prevalence of diabetes mellitus among patients with non-alcoholic fatty liver disease in a tertiary care centre.

## METHODS

A descriptive cross-sectional study was conducted in the Outpatient Department of Internal Medicine at Nepal Medical College and Teaching Hospital (NMCTH) from 1 January 2023 to 29 June 2023 after obtaining ethical approval from the Institutional Review Committee of the hospital (Reference number: 60-079/080). Patients diagnosed with non-alcoholic fatty liver disease based on clinical history including alcohol consumption of 30 grams/day in men and 20 grams/day in women and ultrasonography findings of fatty liver disease at the Internal Medicine department of NMCTH within the study period were included in the study. Patients who had a history of alcohol consumption estimated more than 30 grams/day among males and 20 grams/day among females, and patients with a history of Hepatitis B surface antigen (HBsAg) positive and Anti Hepatitis C Virus (Anti HCV) positive cases were excluded from the study. Convenience sampling was done. The sample size was calculated using the following formula:


$$\begin{array}{ll} \text{n} & ={\text{Z}}^{2}\times \frac{\text{p}\times \text{q}}{{\text{e}}^{\text{2}}}\\ & =1.96^{2}\times \frac{0.240\times 0.759}{0.05^{2}}\\ & =146 \end{array}$$

Where,

n = minimum required sample size
Z = 1.96 at 95% Confidence Interval (CI)
p = prevalence of diabetes mellitus taken as 24.08%^7^
q = 1-p
e = margin of error, 7%

The minimum required sample size was 146. However, 150 samples were included in the study.

Participants were included in this study after they gave written consent for history taking, physical examination and necessary investigations. Data regarding sociodemographic factors, height, weight, history of alcohol consumption and amount of daily alcohol consumption (if any) were taken from all the participants in the study. All the participants measured body mass index (BMI). Non-alcoholic fatty liver disease was diagnosed based on the history and was further graded based on ultrasonography findings as Grade 1 fatty liver for increased liver echogenicity without haziness of vessel walls, Grade 2 fatty liver for increased liver echogenicity with haziness of vessel walls and Grade 3 fatty liver for increased liver echogenicity leading to loss of normal contrast between liver and diaphragm.^5^ The venous blood samples of patients with nonalcoholic fatty liver disease were collected and sent for serology (HBsAg and anti HCV) and blood glucose level investigation (fasting, postprandial and HbA1C). The blood glucose levels were classified based on the American Diabetes Association classification for diagnosis of DM. Blood glucose level of fasting ≥126 mg/dl, postprandial ≥200 mg/dl and HbA1C ≥6.5% was considered a diabetic level.^8^

Data were entered into Microsoft Excel and analysis was performed using IBM SPSS Statistics version 17.0. The point estimate was calculated at a 95% Confidence Interval.

## RESULTS

**Figure 1:**
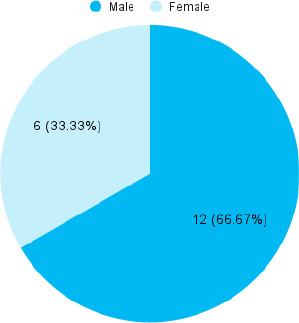
Gender-wise distribution (n= 18)

Among 150 patients with non-alcoholic fatty liver disease, diabetes mellitus was seen in 18 (12%) (6.80-17.20, 95% Confidence Interval). The mean age of the study population was 40.88±11.302 years. In patients with non-alcoholic fatty liver disease, diabetes mellitus was more prevalent in males 12 (67%) (Figure 1).

Fasting glucose levels were increased in the majority of the NAFLD patients with a mean value of 100.22±18.56 mg/dl. Similarly, postprandial glucose levels were increased with a mean value of 151.92±31.43 mg/dl (Table 1).

**Table 1:** Baseline characteristics (n= 18).

Variables	Mean+SD
Age (years)	40.88±11.302
Height (m)	1.609±0.133
Weight (kg)	68.41±10.56
BMI (kg/m^2^)	26.97±6.46
HbA1c (%)	5.46±0.78
Fasting glucose (mg/dl)	100.22±18.56
Postprandial glucose (mg/dl)	151.92±31.43

Among 18 non-alcoholic fatty liver disease patients with diabetes mellitus, most of the patients 11 (61.11%) had a BMI in the range of 23-28 kg/m^2^ (Table 2).

**Table 2:** BMI among non-alcoholic fatty liver disease patients with diabetes mellitus (n= 18).

Age group (years)	n (%)
< 23	4 (22.22)
23-28	11 (61.11)
>28	3 (16.67)

## DISCUSSION

Data were entered into Microsoft Excel and analysis was performed using IBM SPSS Statistics version 17.0. The point estimate was calculated at a 95% Confidence Interval. The prevalence of diabetes mellitus in our study was 18 (12%) out of 150 patients with non-alcoholic fatty liver disease which is lower compared to a previous research study in which the prevalence of diabetes mellitus in non-alcoholic fatty liver disease was found to be 24.08%.^7^ Another study reported prevalence of diabetes mellitus was almost threefold higher (14%) in patients with non-alcoholic fatty liver disease than in patients without non-alcoholic fatty liver disease (5%). In this study, participants with non-alcoholic fatty liver disease had higher BMI in comparison to those without non-alcoholic fatty liver disease.^8^ Similarly, findings in our study revealed increased BMI in patients with NAFLD with a mean of 26.97±6.46 kg/m^2^.

In a research study done in Japan among patients with fatty liver disease, there was a higher incidence of diabetes in males (12.5%) compared to females (26.3%).^9^ In contrast, our study revealed an increased prevalence of diabetes mellitus in males 12 (67%) compared to females 6 (33%) among participants with NAFLD. As for ultrasonography-defined non-alcoholic fatty liver disease (liver steatosis >20%), recent data showed a twofold to fivefold increased risk of diabetes mellitus.^10^ In our study diabetes mellitus was more prevalent in patients with Grade 3 NAFLD 7 (17%) and Grade 1 NAFLD 9 (12%) as compared to Grade 2 NAFLD 2 (5%).

The results of the study cannot be generalised as this study is single institution-based. A multicentre study with a large sample size can be done to achieve more generalised data. Since it is a descriptive crosssectional study, an association between diabetes mellitus and non-alcoholic fatty liver disease could not be made in this study design. Risk factors could not be made out as well. Hence, higher levels of studies are recommended.

## CONCLUSIONS

The prevalence of diabetes mellitus among nonalcoholic fatty liver disease patients was found to be lower than other studies done in a similar setting. The coexistence of diabetes mellitus and non-alcoholic fatty liver disease leads to poor clinical outcomes. Hence, patients with non-alcoholic fatty liver disease must be routinely screened for diabetes mellitus so that an early diagnosis, medical management and lifestyle modifications can be done.

## References

[1] Fletcher B, Gulanick M, Lamendola C (2002). Risk factors for type 2 diabetes mellitus.. J Cardiovasc Nurs..

[2] Byrne CD, Targher G (2015). NAFLD: a multisystem disease.. J Hepatol..

[3] Lomonaco R, Ortiz-Lopez C, Orsak B, Webb A, Hardies J, Darland C (2012). Effect of adipose tissue insulin resistance on metabolic parameters and liver histology in obese patients with nonalcoholic fatty liver disease.. Hepatology..

[4] Petersmann A, Muller-Wieland D, Muller UA, Landgraf R, Nauck M, Freckmann G (2019). Definition, classification and diagnosis of diabetes mellitus.. Exp Clin Endocrinol Diabetes..

[5] Ekstedt M, Franzen LE, Mathiesen UL, Thorelius L, Holmqvist M, Bodemar G (2006). Long-term follow-up of patients with NAFLD and elevated liver enzymes.. Hepatology..

[6] American Diabetes Association Professional Practice Committee. (2022). Classification and Diagnosis of Diabetes: Standards of Medical Care in Diabetes-2022.. Diabetes Care..

[7] Singh SP, Singh A, Pati GK, Misra B, Misra D, Kar SK (2014). A study of prevalence of diabetes and prediabetes in patients of non-alcoholic fatty liver disease and the impact of diabetes on liver histology in Coastal Eastern India.. Journal of Diabetes Mellitus..

[8] Ortiz-Lopez C, Lomonaco R, Orsak B, Finch J, Chang Z, Kochunov VG (2012). Prevalence of prediabetes and diabetes and metabolic profile of patients with nonalcoholic fatty liver disease (NAFLD).. Diabetes Care..

[9] Tokita Y, Maejima Y, Shimomura K, Takenoshita S, Ishiyama N, Akuzawa M (2017). Non-alcoholic fatty liver disease is a risk factor for type 2 diabetes in middle-aged Japanese men and women.. Intern Med..

[10] Gosink BB, Lemon SK, Scheible W, Leopold GR (1979). Accuracy of ultrasonography in diagnosis of hepatocellular disease.. AJR Am J Roentgenol..

